# Fungal Endocarditis: A Case Report

**DOI:** 10.7759/cureus.20156

**Published:** 2021-12-04

**Authors:** Margarida Brito Monteiro, Rita Sismeiro, Catarina Negrão, Marta Jonet, Gonçalo Simoa

**Affiliations:** 1 Internal Medicine, Hospital Prof. Doutor Fernando Fonseca, Amadora, PRT; 2 Cardiology - Ecocardiography, Hospital Prof. Doutor Fernando Fonseca, Amadora, PRT

**Keywords:** embolic phenomena, aortic vegetation, candida parapsilosis, fungal endocarditis, endocarditis

## Abstract

Fungal endocarditis is a rare disease, and it is associated with severe complications and poor prognosis despite combined clinical and surgical treatment. Although *Candida albicans (C. albicans)* is the most common etiological agent of this severe form of endocarditis, *Candida parapsilosis (C. parapsilosis)* is the most common non-albicans causative species. It occurs mostly in patients with predisposing risk factors, and the rarity of this disease demands a high index of suspicion; the diagnosis must be vigilantly pursued by echocardiography and multiple blood cultures. The past few decades have witnessed a rise in the incidence of this disease, mainly due to improvements in the diagnostic approach. We report the case of a 63-year-old man with no previous medical history of cardiac disease and no risk factors who was diagnosed with fungal endocarditis due to *C. parapsilosis* without fungemia. This report illustrates a rare case of fungal endocarditis in a patient with no risk factors and highlights the challenges encountered in the diagnosis, along with complications and predictors of poor prognosis.

## Introduction

Infective endocarditis is a deadly disease. Despite recent improvements in its management, it is still associated with high mortality and serious complications. Fungal etiology accounts for only 2-4% of all infective endocarditis cases [1], and its incidence has increased in the past few decades due to a growing number of patients at risk as well as improved diagnostic methods [2]. It can affect native and prosthetic valves or be cardiac device-related, and it is predominantly associated with host-predisposing conditions, like immunosuppression, or risk factors, such as prosthetic valves, indwelling central venous catheters, prolonged fungemia, or intravenous (IV) drug use. Invasive candida infections are most often associated with candidemia, which primarily occurs in immunocompromised patients and those requiring intensive care. Disseminated fungal infections in immunocompetent individuals are uncommon. Although *Candida albicans (C. albicans)* represents the main etiology of fungal endocarditis, *Candida parapsilosis (C. parapsilosis) is *the predominant subtype of non-albicans species associated with the condition [3].

## Case presentation

A 63-year-old man with a past medical history of hypertension, benign prostatic hyperplasia, and smoking habit presented to the hospital with a three-month history of generalized arthralgia, painful inguinal adenopathies, night sweats, anorexia, and weight loss (8 kg in the past eight months). Increasingly frequent disorientation was also mentioned. He had no history of illicit substances use, dental caries, or any recent invasive procedures or surgeries.

On admission, the patient was febrile (38.3 ºC), had a pulse rate of 83 beats/minute, and blood pressure of 123/74 mmHg. His respiratory rate was 16 breaths/minute with an oxygen saturation of 97% by pulse oximetry on room air. Cardiac auscultation revealed a regular rate and rhythm without murmurs, and lung auscultation was normal. The patient's abdomen was soft without any palpable masses but he presented a 2-cm, soft, mobile, non-painful adenopathy in the right iliac fossa. No other adenopathies were palpable in the remaining ganglionic chains. No other signs were noticed on physical examination, such as Roth spots, Osler nodes, Janeway lesions, or hematuria.

Blood tests revealed raised inflammatory markers (leukocytosis of 12.6 x 10^9^/L and C-reactive protein of 9.45 mg/dL), elevated erythrocyte sedimentation rate (93 mm/hour), anemia (hemoglobin of 9.8 g/dL), and hepatic cholestasis (alkaline phosphatase of 201 U/L, gamma-glutamyl transferase of 169 U/L, and lactate dehydrogenase of 466 U/L). cytomegalovirus, Epstein-Barr virus, and HIV titers were all negative. Urinalysis did not show proteinuria or hematuria and urine sediment was negative. Three sets of blood cultures (each one composed of two aerobic and one anaerobic test) were collected and were found to be negative. Electrocardiogram and non-contrast head CT scan were unremarkable.

**Figure 1:**
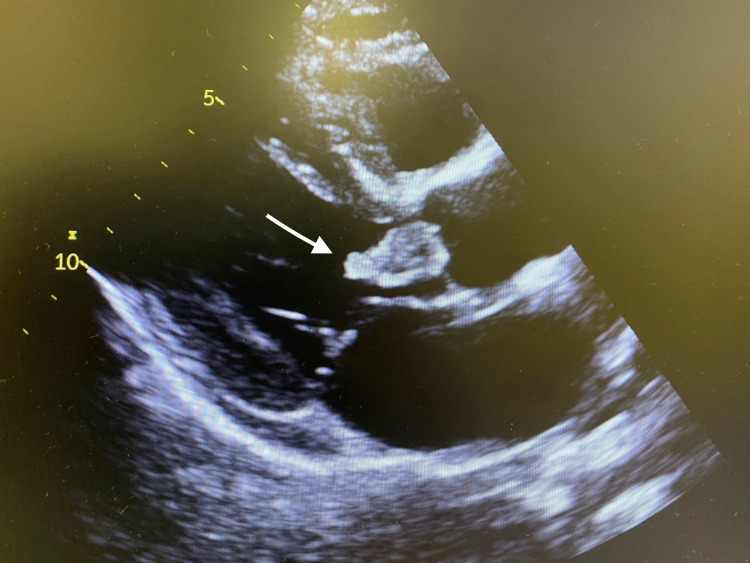
Transthoracic echocardiography (parasternal long-axis view) showing the presence of large vegetation in the aortic valve (arrow)

**Figure 2:**
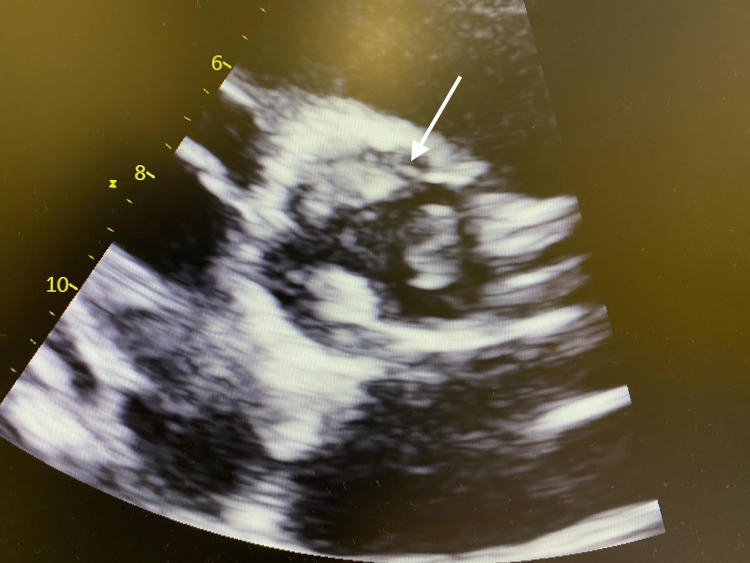
Transthoracic echocardiography (parasternal short-axis view) showing the presence of mass involving the three cups (arrow)

The patient was admitted to the internal medicine ward for further investigation. In the setting of a possible fever of unknown origin (although fever was only noticed after admission), the initial clinical impression was that of lymphoproliferative disease. A thoraco-abdominopelvic CT was performed, which showed moderate heterogeneous splenomegaly and some hypodense areas reflecting probable infarct areas in the spleen and in the kidneys. Suspecting septic embolisms, a diagnosis of endocarditis was considered. A transthoracic echocardiogram (TTE) revealed an extremely large and mobile hyperechoic mass involving the three cusps of the aortic valve with severe valve insufficiency (Figures 1, 2).

The transesophageal echocardiogram (TEE) confirmed the presence of voluminous vegetations at the level of the three aortic valve leaflets (the largest with 20.5 mm) with areas of extraordinary mobility and high embolic risk. The diagnosis of infective endocarditis was made and empirical combined therapy with flucloxacillin, ampicillin, and gentamicin was started. A new set of blood cultures was repeated (two aerobic blood tests) and were positive for *Streptococcus viridans (S. viridans)* sensitive to penicillin, and hence the antibiotic therapy was readjusted, retaining only penicillin and gentamicin.

Given the degree of valve destruction, the patient was referred to a cardiothoracic surgery center and an aortic bioprosthetic valve was implanted. In the postoperative period, the patient presented with dysarthria, left-sided hemiplegia, and left facial droop. A head CT revealed many hypodense areas in the brain compatible with a thromboembolic ischemic stroke. Despite aggressive medical management, the patient, unfortunately, passed away. The result of the microbiological analysis of the removed tissue was only available post-mortem, and *C. parapsilosis* was the only microorganism isolated. Due to the delay in microbiological identification, antifungal therapy had not been started.

## Discussion

*C. parapsilosis* endocarditis is a very rare entity, although its incidence has been on the rise in the last few decades [4]. This agent is predominantly reported in patients with predisposing risk factors, such as prosthetic valves, IV drug use, IV parenteral nutrition, abdominal surgery, immunosuppression, treatment with broad-spectrum antibiotics, and previous valvular disease. In a review involving 72 patients with endocarditis due to *C. parapsilosis*, an absence of predisposing risk factors, as in our patient, was observed in only 6% of cases [2].

The clinical presentation of endocarditis is highly variable and nonspecific: a broad spectrum of symptoms such as fever, dyspnea, chest pressure, asthenia, altered mental status, new cardiac murmur, or signs of acute or chronic heart failure may be present. Peripheral embolic and/or hemorrhagic events are also common, and a severe embolic complication can be the first and only symptom of this disease [3]. In fact, when compared to bacterial endocarditis, fungal endocarditis is associated with a higher incidence of embolic events [2]. The Duke criteria are a set of clinical criteria put forward for the diagnosis of infective endocarditis [5]. In cases of fever of unknown origin, infective endocarditis should be considered and evaluation based on Duke criteria should be performed in all patients. Our patient, for example, presented no major criteria and only one minor criterion at admission and prior to the echocardiogram: fever was noticed but the first three sets of blood cultures collected were negative; moreover, there were no predisposing factors, IV drug use history, or any immunological phenomena. As soon as the body CT scan revealed infarct areas, a TTE was performed, which showed evidence of endocardial involvement with positive echocardiography findings consistent with infective endocarditis.

Subsequent blood cultures isolated *S. viridans*, which, in addition to fever and vascular phenomena, fulfilled the Duke criteria for a definite diagnosis of infective endocarditis (two major plus two minor criteria). However, as in the described case, some common clinical signs and symptoms, like immunological phenomena, may be absent, making this a diagnostic challenge.

Due to the often nonspecific clinical presentation, a correct diagnosis is frequently made only after a long period of variable symptoms, multiple investigations, and long-term hospitalization, as in the reported case. Pathogen detection can be made via blood cultures or be based on the analysis of surgical specimens [1], like in this case. Blood cultures are negative in 2-40% of cases of endocarditis, with some studies reporting blood cultures being negative in up to 71% of cases [6]. For Candida species, blood cultures are positive in less than 50% of cases [7]. Current guidelines for the diagnosis of endocarditis also recommend the culture of valvular tissue, with culture results being used to direct the duration of postoperative antimicrobial therapy [6]. Several studies have shown that the culture of valve tissue suffers from low sensitivity and specificity, with positive cultures identified in only 6-26% of endocarditis cases [6]. Detection of β-d-Glucan, which is an attractive antigen that is found in a broad range of fungal agents, including Candida spp., can also be done, but it is not easily available locally, and some studies have raised concerns about false-positive tests [8]. Echocardiography plays a major role in the detection of fungal endocarditis and should be performed within the first 24 hours. The sensitivity of the echocardiography is limited, especially in prosthetic valve endocarditis, and hence a high index of suspicion is needed to establish the diagnosis, in order to enable early initiation of therapeutic measures that can improve the prognosis. The aortic valve seems to be the most commonly involved valve, with 42.5% of endocarditis cases described occurring in native valves [2].

A therapeutic approach combining antifungal agents and valve replacement surgery should be started immediately since it is thought to offer improved clinical outcomes, although successful outcomes have been reported with medical therapy alone. Surgical intervention is performed in 25-53% of cases of endocarditis [6]. There is no consensus regarding the timing of surgery, but in the case of native valve endocarditis, surgery should be performed within a week [3]. If the microbiological diagnosis has not been established at the time of surgery, the excised valvular tissue should be submitted for histopathological and microbiological evaluation [6]. Unfortunately, in the patient that we describe in this report, microbiological identification was only available post-mortem, which precluded the start of directed antifungal agents.

Despite the recent advances in diagnostic and therapeutic strategies, and even with the use of a combination of medical and surgical therapies, this disease carries high morbidity and mortality risk (mortality rate of about 42%) [2]. Delays in microbial diagnosis may contribute to the delayed initiation of effective antimicrobial therapy, which may lead to high rates of morbidity and mortality.

## Conclusions

This case report provides significant learning points about this infrequent but debilitating type of endocarditis. A high index of suspicion is required for its diagnosis, even in the absence of any apparent risk factors for fungal endocarditis. It is essential to be aware of embolic and immunological phenomena, which can be the only clues to raising infective endocarditis as a diagnostic hypothesis. Proper diagnosis might require serial TTE/TEE, repeated blood cultures, and sometimes it is only achieved with surgical specimen collection, which can prove to be a challenging endeavor. Its treatment usually involves a combination of medical and surgical approaches; nevertheless, it is still associated with extremely high morbidity and mortality, coupled with a much poorer prognosis when compared to bacterial causes. Early detection and treatment are the keys to lower morbidity and mortality.
